# A bibliometric analysis of health-related literature on natural disasters from 1900 to 2017

**DOI:** 10.1186/s12961-019-0418-1

**Published:** 2019-02-11

**Authors:** Waleed M. Sweileh

**Affiliations:** Department of Physiology, Pharmacology/Toxicology, Division of Biomedical Sciences, College of Medicine and Health Sciences, An-Najah National University, Nablus, Palestine

**Keywords:** Natural disasters, health, bibliometric analysis

## Abstract

**Background:**

Worldwide, natural disasters have caused a large number of deaths and considerable morbidity. Nevertheless, limited information is available on how the health-related literature on natural disasters has evolved. The current study aims to assess the growth and pattern of health-related literature on natural disasters.

**Method:**

A bibliometric method was implemented using Scopus database for the period from 1900 to 2017. Keywords used in the search strategy were obtained from the classifications of natural disasters presented by the Centre for Research on the Epidemiology of Disasters. The health component was determined by selecting the health-related subject areas in Scopus.

**Results:**

In total, 9073 documents were retrieved. The annual number of publications showed a noticeable sharp increase after 2004. The retrieved documents received 97,605 citations, an average of 10.8 per document. The *h*-index of the retrieved documents was 113. Author keywords with the highest occurrence were ‘earthquakes’ followed by ‘disaster medicine’, ‘disaster planning’, ‘tsunami’, ‘mental health’, ‘disaster preparedness’, ‘PTSD’, ‘emergency preparedness’, and ‘public health’. Authors from the United States of America contributed to 3127 (34.5%) publications and ranked first, followed by those from Japan (700; 7.7%) and China (636; 7.0%). When research output was standardised by Gross Domestic Product per capita, India ranked first, followed by China and the United States. The United Kingdom had the highest percentage of documents with international authors, followed by those from Switzerland and Canada. The *Prehospital and Disaster Medicine* journal published the most articles (636; 7.0%). The Sichuan University and its affiliated hospital contributed to 384 (7.0%) documents and ranked first in the field.

**Conclusion:**

The current baseline information on health-related literature on natural disasters showed that this field is growing rapidly but with inadequate international research collaboration. Research collaboration in this field needs to be strengthened to improve the global response to natural disasters in any place in the world. There is a need to expand the research focus in this field to include communicable and non-communicable diseases. Finally, the health effects of other natural disasters, such as floods, droughts and disease outbreaks, need to be addressed.

**Electronic supplementary material:**

The online version of this article (10.1186/s12961-019-0418-1) contains supplementary material, which is available to authorized users.

## Background

According to the Centre for Research on the Epidemiology of Disasters (CRED) [1], a disaster is defined as “*an unforeseen and often sudden event that causes great damage, destruction and human suffering*” [2]. Natural disasters are defined as “*naturally occurring physical phenomena caused either by rapid or slow onset events which can be geophysical, hydrological, climatological, meteorological or biological*” [2, 3]. In the last decade, more than 2.6 billion people have been affected by natural disasters [4]. The disasters of hydrometeorological origin are the most recurrent and dangerous type [3]. According to CRED, the number of earthquakes worldwide has increased roughly four-fold in the past 25 years [3]. Furthermore, natural disasters usually lead to mass destruction that can overwhelm national medical resources and prevent the delivery of comprehensive and definitive medical care to affected people in affected areas [5–11].

The major advancements in communications and telecommunications have interconnected most regions of the planet, thus facilitating rescue processes and health responses in even the most distant regions following natural disasters such as devastating earthquakes, tsunamis or the sudden emergence of new widespread infectious diseases. The health response to a large-scale natural disaster becomes extremely important when such disasters occur in low- and middle-income countries where resources are limited and the number of trained human staff is inadequate. A poor health response to massive natural disasters might negatively affect global health because of the potential emergence of serious and unconfined communicable diseases and the emergence of many people seeking shelter, with subsequent social destabilisation.

The 2030 agenda for Sustainable Development Goals and Sendai Framework for Disaster Risk Reduction intend to reduce the number of lives lost and minimise human suffering from natural disasters across the globe [12–14]. Therefore, research collaboration and networking to increase national preparedness and management of natural disasters is of great importance. Such research collaboration aims to strengthen global health by minimising the health consequences of natural disasters worldwide. One method to gain an extensive and comprehensive understanding of this emerging field is an application of bibliometric analysis to health-related literature on natural disasters. Such bibliometric analysis could strengthen and enhance contributions of scholars to this emerging field by identifying the research gaps at the global level. Bibliometric analysis on man-made disasters, such as conflicts and refugees, have been published [15]. However, to the best of the author’s knowledge, no bibliometric analysis of health-related literature on natural disasters is available to date. Therefore, the objectives of this study were (1) to quantify and assess the health-related literature on the field of natural disasters, (2) to map the health-related literature and the extent of research collaboration in natural disasters, and (3) to identify the most important health-related issues pertaining to the literature in natural disasters. Such objectives will answer the following questions: What is the general state of health-related literature on natural disasters? What are the most commonly used author keywords and terms in this field (1968–2017)? What countries, institutions and journals are the most actively involved in this field?

## Methods

### Bibliometric versus systematic review methodology

A bibliometric analysis was performed using the SciVerse Scopus database. Bibliometric analyses are different from systematic reviews or scoping reviews [16–19]. In systematic reviews, several databases are used to retrieve literature in a certain subject and the retrieved literature is then filtered based on predetermined inclusion and exclusion criteria to obtain a limited number of articles. In many cases, these filtered articles are statistically analysed (meta-analysis) to obtain new data. In scoping reviews, different databases are also searched and articles are retrieved and filtered. The filtered literature is usually limited in number and analysed in terms of study designs used in the retrieved documents. Conversely, in bibliometric analysis, a large database, such as SciVerse Scopus, is used to retrieve, analyse and map the data. In addition, bibliometric analysis provides information about citations and research collaboration.

### The database used

In the current study, Scopus was used because it is 100% inclusive of MEDLINE. Secondly, Scopus has a larger number of indexed journals (approximately 23,000 journals) than Web of Knowledge. Therefore, the volume of literature retrieved from Scopus will be larger than that obtained from Web of Science [20]. Thirdly, Scopus has many functions that facilitate citation analysis, counting research collaboration, and data export to Microsoft Excel for further tabulation and mapping. Indeed, many published bibliometric studies have used Scopus as the tool to retrieve the required data [21, 22].

### The search strategy

The search strategy utilised specific keywords used in title/abstract with certain constraints. We also used journal names such as ‘disaster’ or ‘emergenc*’ or ‘natural hazard’ to retrieve documents related to natural disasters (Additional file 1). Keywords used in the search strategy were obtained from websites pertaining to disasters such as CRED and Red Cross [1, 23]. For the health component of the study, we used a function in Scopus, which categorises the retrieved documents into subject areas. In the current study, only documents in the following subjects were retrieved: medicine, nursing, psychology, biochemistry/molecular biology, neuroscience, pharmacology, health professions and veterinary science. Thus, documents about natural disasters published within the subject area of health were retrieved. Some keywords might retrieve irrelevant or false-positive results, for which an exclusion step was implemented. The exclusion step included, but was not limited to, keywords such as ‘world trade centre’ or ‘trade centre’ , ‘terrorist attack’ , ‘sarin attack’ and others. The excluded keywords were obtained by searching the retrieved documents, particularly those in the top 1000 cited documents. In the search strategy, an asterisk was used to retrieve as many numbers of related keywords as possible. The quotation marks were used to retrieve the exact phrases written in the search strategy.

### The validation of the search strategy

The search strategy was modified several times to sharpen the results and increase the validity of the search strategy. The modifications were made to ensure minimum false-positive and false-negative results. To ensure minimum false-positive results, the top 1000 cited documents were reviewed to ensure that they fell within the scope of the study. For false-negative results, the number of documents for the top active authors shown in the Scopus database was compared with their research profile in Scopus to assess the extent of agreement between what has been retrieved and what is actually in the Scopus database pertaining to the desired research question. The extent of agreement was tested using interclass correlation test in SPSS [24–28]. The results obtained showed that the search strategy had minimum (insignificant) false-negative results.

### Bibliometric indicators and mapping

In any bibliometric analysis, retrieved data are analysed to generate specific indicators such as annual growth of publications, most frequent author keywords to gain insight into research interest and research gaps, calculation of international research collaboration, and most active authors, journals, countries and institutions. The research output was standardised by Gross Domestic Product (GDP) and population size. The GDP per capita for each country was obtained from World Bank data [29].

For calculation of international collaboration, the number of documents with authors having different country affiliations was counted for each country. The number of documents with no international research collaboration was counted by deducting the number of documents with international collaboration from the total number of documents counted for each country. In the current study, publications with international collaboration were termed ‘multiple country publications’ while publications with no international collaboration were termed ‘single country publications’. Research collaboration was mapped using VOSviewer software, a free online programme available for download from Leiden University [30, 31]. In VOSviewer mapping, both the thickness of the connecting line and the distance between any two nodes (countries) reflect the extent of research collaboration. When using the VOSviewer to map most frequent author keywords, the size of the node of the keyword represents the frequency of appearance of that keyword. Geographical mapping of the number of publications was carried out using ArcMap 10.1. In geographical mapping, the country affiliation of each author in the retrieved documents was used to determine the extent of contribution for each country. For citation analysis, the Hirsh-index (*h*-index) was used as a measure of both the number of publications and the number of citations they received, with a higher *h*-index suggesting a higher impact [32]. The *h*-index could be used for countries, journal, authors or any set of publications.

## Results

### Volume of the retrieved literature

The total number of health-related documents on natural disasters was 9073. When the search strategy was used without restriction to health subject areas, approximately 60,000 documents were retrieved. Therefore, the health component of literature on natural disasters accounted for less than 16%. The remaining literature focused on earth sciences, engineering, environment and other areas. In total, 23,374 authors contributed to publishing the retrieved documents, yielding an average of 2.6 authors per document.

### Types of documents and languages

Analysis of the retrieved documents showed that 6941 (76.5%) were research articles and 1031 (11.4%) were review articles. Other types of documents included editorials (250; 2.8%), notes (237; 2.6%), conference papers (208; 2.3%), letters (208; 2.3%) and short surveys (68; 0.7%). The vast majority of the retrieved documents were in English (7853; 86.6%), with other languages including Chinese (308; 3.4%), Japanese (226; 2.5%), German (168; 1.9%), French (149; 1.6%), Spanish (105; 1.2%) and Russian (95; 1.0%).

### Annual growth of publications

**Fig. 1:**
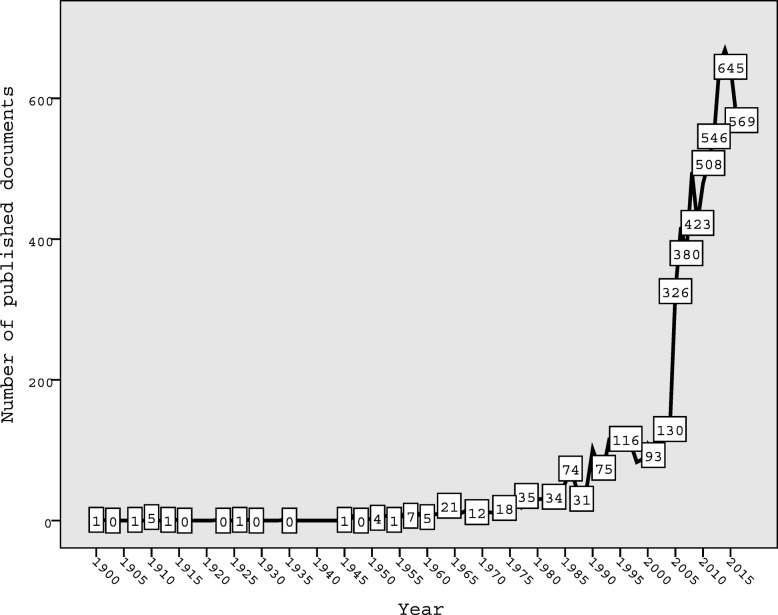
Annual growth of health-related publications on natural disasters (1900–2017)

Figure 1 shows the annual growth of publications. The graph shows that the number of publications remained low until early 1990, followed by a steady phase until 2004. There was a noticeable sharp increase in the number of publications after 2004, which reached a maximum of approximately 600 publications per year in the last 5 years of the study period.

### Citation analysis

The retrieved documents received 97,605 citations, an average of 10.8 per document. The *h*-index of the retrieved documents was 113. The range of citations was from 0 to 538. The article that received the highest citations was published in 1996 in *New England Journal of Medicine* [33]. Table 1 shows the top cited documents during the study period. Half of the documents were published in psychiatry/psychology journals. Four articles were on the health-related issues of earthquakes, one focused on floods, one on a cholera outbreak, one on hurricanes, and one on climate change. The remaining articles had a general focus.

**Table 1:** Top 10 cited documents on health-related aspects of natural disasters (1900–2017)

Title	Year	Journal name	Number of citations	Type of document
Sudden cardiac death triggered by an earthquake	1996	*New England Journal of Medicine*	531	Article
Post-traumatic stress disorder following disasters: A systematic review	2008	*Psychological Medicine*	411	Review
The origin of the Haitian cholera outbreak strain	2011	*New England Journal of Medicine*	395	Article
Post-traumatic stress reactions in children after the 1988 Armenian earthquake	1993	*British Journal of Psychiatry*	366	Article
Climate change and extreme heat events	2008	*American Journal of Preventive Medicine*	325	Review
Psychiatric comorbidity in children after the 1988: earthquake in Armenia	1995	*Journal of the American Academy of Child and Adolescent Psychiatry*	300	Article
Global health impacts of floods: epidemiologic evidence	2005	*Epidemiologic Reviews*	298	Article
The serotonin transporter genotype and social support and moderation of posttraumatic stress disorder and depression in hurricane-exposed adults	2007	*American Journal of Psychiatry*	278	Article
Children exposed to disaster: I. Epidemiology of post-traumatic symptoms and symptom profiles	1994	*Journal of the American Academy of Child and Adolescent Psychiatry*	273	Article
Dissociative reactions to the San Francisco Bay Area earthquake of 1989	1993	*American Journal of Psychiatry*	273	Article

### Most frequent author keywords

**Fig. 2:**
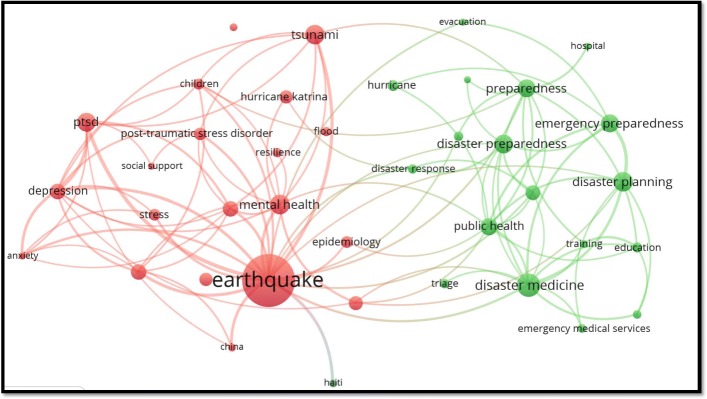
Network visualisation map of author keywords of health-related literature on natural disasters (1900–2017)

Figure 2 shows a network visualisation map of author keywords with minimum occurrences of 30. Author keywords with the highest number of occurrences were ‘earthquakes’ followed by ‘disaster medicine’ , ‘disaster planning’ , ‘tsunami’ , ‘mental health’ , ‘disaster preparedness’ , ‘PTSD’ , ‘emergency preparedness’ and ‘public health’. The most occurring author keywords existed within two large clusters, the first including keywords related to earthquakes and psychological and mental health aspects, and the second including author keywords related to disaster medicine, management, preparedness, response, training and education.

### Most active countries

**Fig. 3:**
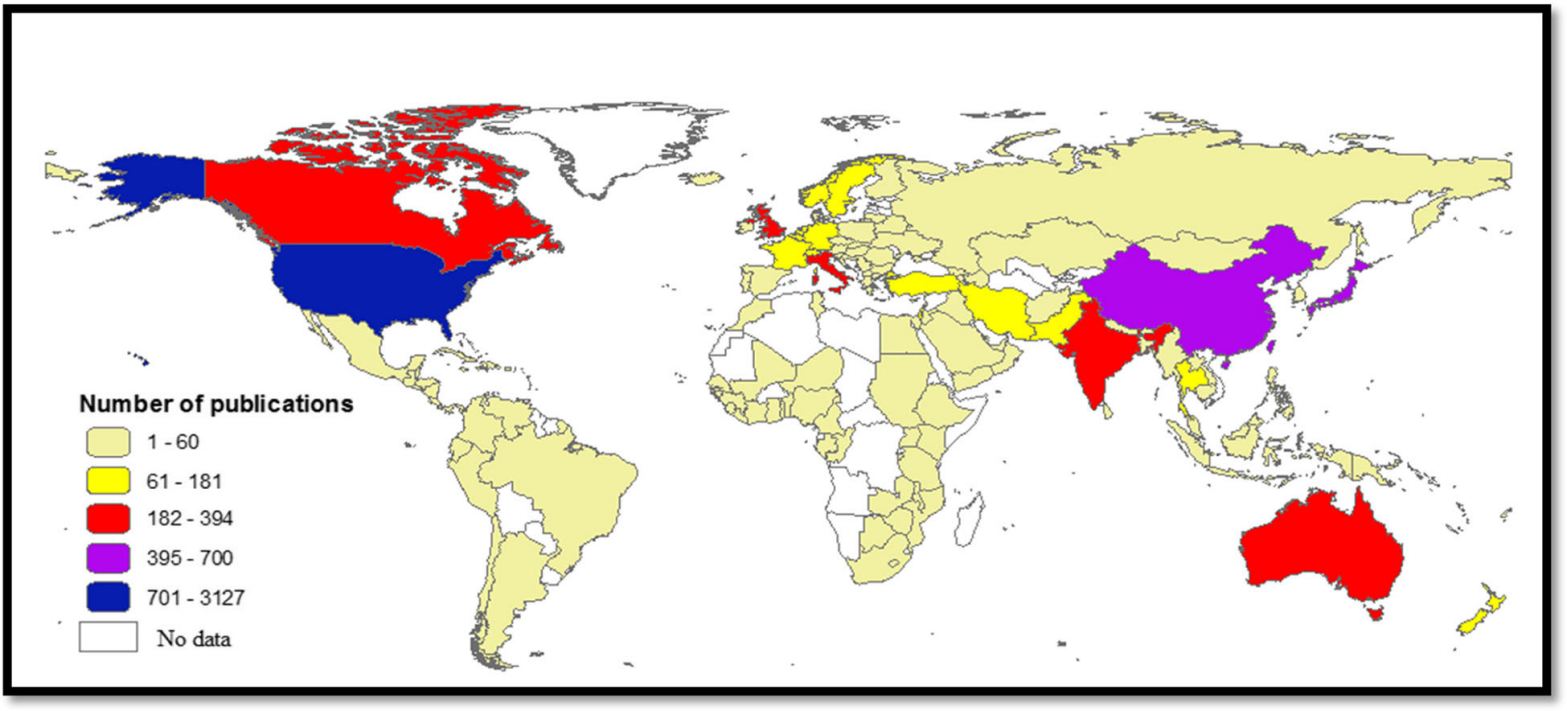
Geographical distribution of health-related literature on natural disasters (1900–2017)

Table 2 shows a list of the 17 countries that have contributed at least 100 publications. Authors from the United States of America contributed to 3127 (34.5%) publications, followed by those from Japan (700; 7.7%) and China (636; 7.0%). When research output was standardised by GDP per capita, India ranked first, followed by China and the United States of America. The geographical distribution of the retrieved documents is presented in Fig. 3. Publications in this field were published from most world regions. However, the North America and Western Pacific regions contributed the most.

**Table 2:** Most active countries and international collaboration on health-related aspects of natural disasters (1900–2017)

Country	Frequency *N* = 9073	%	GDP per capita (×1000)	Number of publications per GDP per capita	Number of collaborating countries	SCP	%	MCP	%
United States	3128	34.5	57.467	54.4	101	2516	80.4	612	19.6
Japan	700	7.7	38.895	18.0	52	568	81.1	132	18.9
China	636	7.0	8.123	78.3	41	466	73.3	170	26.7
Australia	394	4.3	49.928	7.9	52	227	57.6	167	42.4
United Kingdom	357	3.9	39.899	8.9	71	159	44.5	198	55.5
Canada	241	2.7	42.158	5.7	54	117	48.5	124	51.5
Italy	213	2.3	30.527	7.0	47	117	54.9	96	45.1
India	209	2.3	1.741	120.0	29	169	80.9	40	19.1
Germany	181	2.0	41.936	4.3	44	122	67.4	59	32.6
France	161	1.8	36.855	4.4	57	94	58.4	67	41.6
Turkey	152	1.7	10.788	14.1	26	89	58.6	63	41.4
New Zealand	150	1.7	39.427	3.8	9	121	80.7	29	19.3
Iran	138	1.5	5.305	26.0	12	97	70.3	41	29.7
Sweden	123	1.4	51.600	2.4	36	62	50.4	61	49.6
Israel	108	1.2	37.293	2.9	29	58	53.7	50	46.3
Switzerland	108	1.2	78.813	1.4	41	51	47.2	57	52.8
Taiwan	101	1.1	24.577	4.1	21	63	62.4	38	37.6

*GDP* gross domestic product, *SCP* single country publications, *MCP* multiple country publications

### International collaboration

**Fig. 4:**
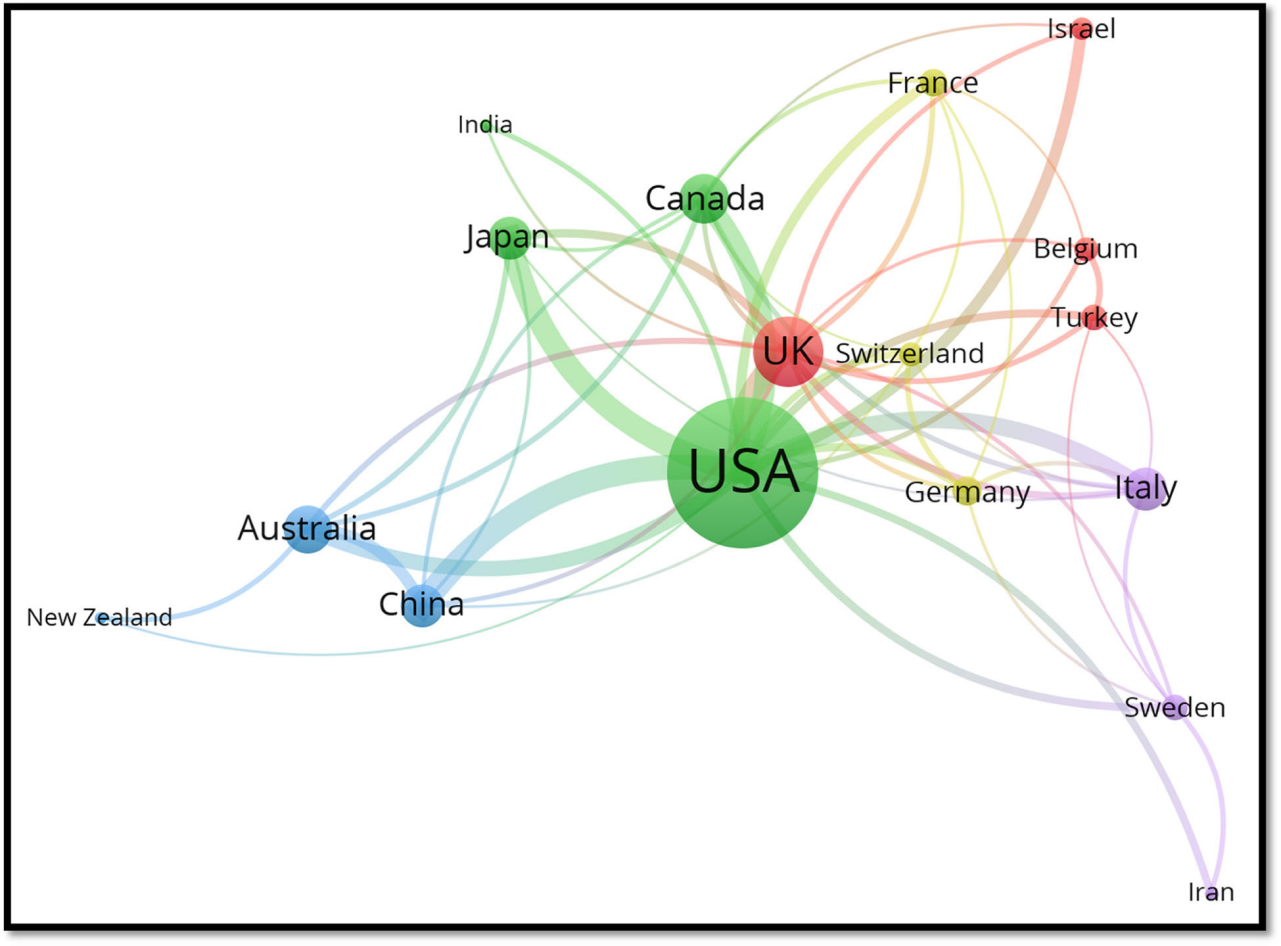
Network visualisation map of health-related literature on natural disasters (1900–2017); only active countries were shown in the map

Figure 4 shows a visualisation map of research collaboration among countries with a minimum contribution of 100 publications. The thickness of the line between any two countries represents the strength of research collaboration between the two countries. The distance between countries reflects how much any two countries are related with respect to this field. For example, Iran and India are not related in this field because there is no connecting line between the two countries and the distance between them is relatively long. China and Australia, on the other hand, are related to each other since they exist in close proximity to each other, with a connecting line between them. The research collaboration between the United States and China was the strongest (link strength = 70), followed by those between the United States and Japan (link strength = 65), and between the United States and the United Kingdom (link strength = 61). The United States had the largest number of collaborating countries, and it therefore occupies the center of the map with many connecting lines with different countries. However, when international collaboration was calculated as a percentage of the total research output for each country, the United Kingdom had the highest percentage of documents with international authors followed by those from Switzerland and Canada (Table 2).

### Most active journals and institutions

The *Prehospital and Disaster Medicine* journal published the most articles (636; 7.0%), followed by *Disaster Medicine and Public Health Preparedness* (619; 6.8%) and *American Journal of Disaster Medicine* (140; 1.5%). Top active journals are shown in Table 3. Six of the top active journals are based in the United States, three are based in the United Kingdom, and one is based in China. The total number of documents published by top 10 active journals was 1928, equivalent to 21.8% of the retrieved documents. Sichuan University and its affiliated hospital contributed to 384 (7.0%) documents and ranked first in the field. The top 10 active institutions/organisations are shown in Table 4. Eight of the top 10 active institutions/organisations are based in the United States, one is based in China, and one is based in Japan.

**Table 3:** Top 10 active institutions on health-related aspects of natural disasters (1900–2017)

Rank	Institution/Organisation	Frequency *N* = 9073	%	Country
1	Sichuan University/West China Hospital of Sichuan University	384	4.2	China
2	Harvard University	209	2.3	United States
3	Johns Hopkins University	206	2.3	United States
4	Centers for Disease Control and Prevention	204	2.2	United States
5	Tohoku University School of Medicine	204	2.2	Japan
6	Tulane University	99	1.1	United States
7	VA Medical Centers	98	1.1	United States
8	University of California, Los Angeles	94	1.0	United States
9	Columbia University in the City of New York	80	0.9	United States
10	Uniformed Services University of the Health Sciences	77	0.8	United States

**Table 4:** Top 10 active journals on health-related aspects of natural disasters (1900–2017)

Rank	Journal	Frequency *N* = 9073	%	Country
1	*Prehospital and Disaster Medicine*	636	7.0	United States
2	*Disaster Medicine and Public Health Preparedness*	619	6.8	United Kingdom
3	*American Journal of Disaster Medicine*	140	1.5	United States
4	*Chinese Journal of Evidence-Based Medicine*	112	1.2	China
5	*Annals of Emergency Medicine*	90	1.0	United States
6	*Journal of Traumatic Stress*	90	1.0	United States
7	*International Journal of Emergency Management*	89	1.0	United Kingdom
8	*American Journal of Public Health*	69	0.8	United States
9	*PLoS Currents*	68	0.7	United States
10	*Lancet*	67	0.7	United Kingdom

## Discussion

In the current study, we investigated the growth and pattern of health-related literature on natural disasters using the Scopus database.

### Volume and growth of publications

In 2004 and 2005, two major and destructive natural disasters hit the United States and the South East Asia and evoked a large number of publications related to natural disasters. The 2004 Indian Ocean earthquake occurred off the west coast of Sumatra, Indonesia, and is the third-largest earthquake ever recorded on a seismograph [34]. The event is known as the Sumatra–Andaman earthquake and is considered the worst in terms of the number of fatalities, prompting a worldwide humanitarian response [35]. Another natural event that evoked a large number of publications was hurricane Katrina, which is one of the deadliest hurricanes to hit the United States [36]. The hurricane prompted a wide international response to help the United States government manage the massive destruction and the large number of displaced people [37, 38]. A third major natural disaster that occurred in South East Asia and prompted a large number of publications was the 2008 Sichuan earthquake or Wenchuan earthquake, which occurred in China in 2008 and left over 69,000 fatalities and at least 4.8 million people homeless [39–41]. A study showed that the number of quake-related medical literature increased after the Wenchuan earthquake [42]. The finding that the retrieved documents had an *h*-index of 113 is a positive indicator of the high interest of researchers and policy-makers in the subject. The *h*-index was high [15, 43] and suggested that a wide range of readers are interested in this topic.

### Most frequent keywords and terms

Visualisation of author keywords showed that the mental health-related literature dominated this field, which is unsurprising given the psychological trauma and loss of loved ones after natural disasters [44–46]. However, there were research gaps in other important fields such as maternal, child and geriatric health; these groups of people are highly important since health services and health systems are severely disrupted in affected areas [47–49]. Published studies showed that natural disasters affect the physical and mental functionalities of older adults and increase their risk of mortality [50–52].

The current study showed that health aspects of earthquakes, hurricanes and tsunamis were the most studied. However, other types of natural disasters, such as floods and droughts, were under-researched. Furthermore, biological natural disasters leading to serious outbreaks and epidemics such as cholera and diarrheal diseases were also under-represented as a category of natural disasters. Potential disease spread and outbreaks in disaster areas are high and evidence-based research on this topic needs to be considered [53, 54]. Evidence-based research helps to shape health management and practices during natural disasters.

### Most active countries, institutions and journals

Data on natural disasters identified China, the United States, the Philippines, Indonesia and India as the five countries most frequently hit by natural disasters [55]. In 2014, of all regions on Earth, Asia had the worst share of natural disasters [55]. Of the worst five countries in terms of natural disasters, only three were among the top active list, while Philippines (28 publications) and Indonesia (51 publications) ranked in the 25th and 36th positions. Research activity is dependent on the number of scholars and academics as well as on the volume of funding available for research. The most active list included countries such as Turkey and Iran. High death numbers were recorded in two major earthquakes in Iran in 1990 and 2003 [56–58]. A bibliometric study showed that Iran ranked 23rd in the number of publications on earthquake research [59]. For Turkey, a bibliometric study showed that the contribution and the number of citations of articles published from Turkey on earthquakes increased significantly after the Marmara earthquake [60].

The findings that most active institutions are located in the United States and Asia reflects the long history of deadly natural disasters in these regions. The current study showed that the extent of international research collaboration was relatively low when compared with international research collaboration in other fields such as malaria and tuberculosis [61–63]. Furthermore, collaboration was of a regional nature rather than a global one. The unpredictable nature and the mass destruction produced by natural disasters require greater international research collaboration, particularly between countries with high scientific and financial capabilities and countries with high vulnerabilities and limited resources. Research collaboration is extremely important for some countries in South America, Africa, Eastern Europe and Asia, which lack health experts and scholars in public health, disaster medicine, emergency medicine and other related fields.

The list of most active journals and institutions/organisations was skewed toward the United States. This could be due to advanced scientific and research capabilities in the United States and the number of natural hazards that affected the United States in the past few decades. Asia is one of the most vulnerable regions to natural disasters. However, the number of retrieved articles from Asian countries was relatively low, likely due to the presence of a large number of unindexed journals published from Asian countries. The number of natural disasters that hit China made Sichuan University one of the top active institutions in the field. According to the International Disaster Database, China experienced a high number of natural disasters in the past few decades [64]. A published study from China showed that the effort of producing and disseminating Wenchuan (China) earthquake-related medical research was the largest in the number of quake-related medical papers in human history [42]. A published systematic review concluded that, from 1980 through 2009, there were 372,634 deaths and over 61 million people affected by earthquakes, and mortality was greatest in Asia [65].

### Strengths and limitations

The current study, to the best of the author’s knowledge, is the first bibliometric study to analyse the health-related literature on natural disasters. Previously published bibliometric studies on natural disasters discussed the literature on a particular type of natural disasters or on natural disasters in a certain region [42, 59, 60, 66]. The health aspect of natural disasters is an emerging and important health component since natural disasters can hit anywhere and the international community needs to be ready for such mass problems, particularly when mixed with poverty and disease outbreaks.

The current study has a few limitations inherent in bibliometric methodology. The presence of false-positive and false-negative results is a possibility in any bibliometric study. The literature in the Scopus database is skewed toward English journals and therefore documents in Chinese, Japanese and other languages were underestimated. Unfortunately, in bibliometric analyses, only one database can be used because data from more than one database cannot be combined and analysed. This is in contrast to systematic reviews, where several databases are used to retrieve a number of documents for further analysis. Furthermore, in the current study, the number of citations and *h*-index were used to assess quality despite their inherent limitations such as the impact of self-citations on the overall number of citations and *h*-index. Finally, in the current study, only documents in peer-reviewed literature were retrieved and analysed while documents in grey literature were not retrieved.

## Conclusion

This study assessed the health-related literature on natural disasters, providing a baseline of information for future research. The retrieved literature focused mainly on the psychological and mental health of individuals. Thus, there is a serious need to study the non-mental health aspects of natural disasters, particularly the potential emergence of serious communicable diseases. The current study showed inadequate international research collaboration in this field. Establishing global research networks that include low- and middle-income countries is important for the future. Such networks will help vulnerable and poor countries to improve their research agenda and preparedness in response to natural disasters. Such collaboration will also create a global platform for experts to exchange information and health lessons and experiences to be learned from natural disasters.

## Additional file

Additional file 1: Search strategy and keywords for retrieving documents in natural disasters health research using Scopus. (DOCX 12 kb)

## Abbreviation

CRED: Centre for Research on the Epidemiology of Disasters
