# Supplementary material for: A bibliometric analysis of health-related literature on natural disasters from 1900 to 2017
Source: Health Res Policy Syst. 2019 Feb 11;17:18. doi: 10.1186/s12961-019-0418-1 (PMC6371570; doi:10.1186/s12961-019-0418-1)
Supplement: Supplementary file 1 — Search strategy and keywords for retrieving documents in natural disasters health research using Scopus. (DOCX 12 kb) [file 12961_2019_418_MOESM1_ESM.docx]

**Additional file 1**

Search strategy and keywords for retrieving documents in natural disasters health research using Scopus

(( ( TITLE ( outbreak ) AND TITLE-ABS ( disaster OR emergenc* OR "mass causa*" OR "biolog* hazard" OR "avian flu" OR cholera OR "dengue fever" OR "ebola" OR "marburg" OR malaria OR measles OR "yellow fever" OR "meningitis" OR tuberculosis OR hiv OR aids ) AND SRCTITLE ( infetc* OR disaster OR health OR medi* ) AND TITLE-ABS ( epidemic ) ) )

**OR**

( ( ( ( SRCTITLE ( "disaster*" OR "natural hazard" OR "emergency" ) AND TITLE ( avalanche* OR "Tidal wave*" OR hurricane OR earthquake OR tsunami OR volcan* OR "cyclo* storm" OR "tropical storm" OR flood OR wildfire OR tornado OR landslide OR sinkhole OR "extreme heat" OR mudslide OR "extreme temperature" OR outbreak OR "ground movement" OR "ash fall" OR "rock fall" OR "lahar" OR "severe storm" OR "heavy storm" ) )

**OR**

(TITLE (disaster*) AND TITLE-ABS (natural OR hurricane OR earthquake OR tsunami OR volcan* OR " cyclo* storm*" OR "tropical storm" OR "heavy storm" OR flood OR wildfire*))

**OR**

TITLE-ABS ( "disaster relief" OR "disaster prepared*" OR "disaster emergen*" OR "disaster management" OR "disaster preparation" OR "disaster planning" OR " disaster prevention" OR "disaster medicine" ) OR ( TITLE-ABS ( health OR "natural disaster*" OR "natural hazard" OR emergency OR "mass causal*" OR psych* OR mental OR stress ) AND TITLE ( avalanche* OR "Tidal wave*" OR hurricane OR earthquake OR tsunami OR volcan* OR "cyclo* storm" OR "tropical storm" OR "heavy storm" OR flood OR wildfire OR tornado OR landslide OR sinkhole OR "extreme heat" OR mudslide OR "extreme temperature" OR mudslide OR tornado* OR avalanche* OR "tidal wave*" ) )

**OR**

(TITLE (drought) AND TITLE-ABS (hunger OR nutri* OR disease OR health))

**AND NOT**

TITLE ( "Necrotizing fasciitis" OR air OR aviation ) ِand AND NOT TITLE ( "air disaster" OR aviation ) AND NOT TITLE ( gut OR "world trade" OR "sarin" OR air OR aviation OR war OR "terrorist attack" OR terrorism ) ِand AND NOT TITLE ( "air disaster" OR aviation OR "financial disaster" ) AND NOT SRCTITLE ( "surgery" OR gut OR pathogen ) AND NOT TITLE ( *virus ) AND NOT TITLE ( necrotizing ) AND NOT TITLE ( soybeans OR "sarin attack" OR *rna OR "techno* disaster" OR "manmade disaster" OR heroin ) ) ) ) AND NOT TITLE ( "world trade center" OR "trade center" ) AND NOT TITLE(terror*)

**AND** **( LIMIT-TO** ( SRCTYPE,"j " ) ) AND ( EXCLUDE ( DOCTYPE,"er " ) )

**AND ( EXCLUDE** ( SUBJAREA,"EART " ) OR EXCLUDE ( SUBJAREA," SOCI " ) OR EXCLUDE ( SUBJAREA," ENGI " ) OR EXCLUDE ( SUBJAREA," ENVI " ) OR EXCLUDE ( SUBJAREA," BUSI " ) OR EXCLUDE ( SUBJAREA," COMP " ) OR EXCLUDE ( SUBJAREA," AGRI " ) OR EXCLUDE ( SUBJAREA," ARTS " ) OR EXCLUDE ( SUBJAREA," ECON " ) OR EXCLUDE ( SUBJAREA," MATH " ) OR EXCLUDE ( SUBJAREA," ENER " ) OR EXCLUDE ( SUBJAREA," DECI " ) OR EXCLUDE ( SUBJAREA," MULT " ) OR EXCLUDE ( SUBJAREA," PHYS " ) OR EXCLUDE ( SUBJAREA," MATE " ) OR EXCLUDE ( SUBJAREA," CENG " ) OR EXCLUDE ( SUBJAREA," CHEM " ) OR EXCLUDE ( SUBJAREA," Undefined " ) )

**AND ( EXCLUDE** ( PUBYEAR,2018 ) ) )
